# Spectrum of *SCN8A*-Related Epilepsy

**DOI:** 10.15844/pedneurbriefs-29-2-7

**Published:** 2015-02

**Authors:** Lindsey A. Morgan, John J. Millichap

**Affiliations:** 1 Division of Neurology, Ann & Robert H. Lurie Children's Hospital of Chicago, Chicago, IL; 2 Departments of Pediatrics and Neurology, Northwestern University Feinberg School of Medicine, Chicago, IL

**Keywords:** Epileptic Encephalopathies, Voltage Gated Sodium Channels, SCN8A, Dravet Syndrome

## Abstract

Investigators from the EuroEPINOMICS European research consortium studied 17 patients with epileptic encephalopathy due to *SCN8A* mutations and reported the specific genetic and phenotypic features.

Investigators from the EuroEPINOMICS European research consortium studied 17 patients with epileptic encephalopathy due to *SCN8A* mutations and reported the specific genetic and phenotypic features. Sixteen mutations were *de novo* and one was inherited from an unaffected somatic mosaic parent. The pathogenic mutations were distributed throughout the entire *SCN8A* gene and 16 were missense. Patients ranged in age from 8 months to 44 years (mean 8 years) at diagnosis and 12 were female. Seizure onset occurred at a mean of 5 months (range 1 day to 18 months). Seizure semiology at onset was variable and included focal clonic seizures evolving to bilateral convulsions, tonic seizures, epileptic spasms, and myoclonic seizures. Most patients developed a second seizure type. Eight patients had status epilepticus. All patients had refractory epilepsy but a minority (n=4) had extended seizure-free periods (range 6 months to 17 years). Development ranged from normal with plateau or regression after seizure onset to abnormal development from birth. All older than 18 months had some intellectual disability ranging from mild to severe. Other neurologic features included hypotonia, dystonia, hyperreflexia, choreoathetosis, and ataxia. Two patients died early in childhood. Brain MRI studies were normal at onset in 9, abnormal in 4 (cerebral atrophy and hypoplasia of the corpus callosum), or not available (n=4). Of the 14 patients with available EEG at seizure onset, it was either normal or had focal or multifocal epileptiform activity. Fifteen patients developed an abnormal EEG with moderate to severe background slowing, and focal or multifocal sharp waves or spikes mostly in temporal region. Sodium channel blockers were effective for 4 patients. [1]

COMMENTARY. Voltage gated sodium channels (VGSC) are integral membrane proteins essential for normal neurologic function and are the most recognized cause of genetic epilepsy [2]. The adult brain has 4 main subtypes of VGSC encoded for by genes *SCN1A*, *SCN2A*, *SCN3A*, and *SCN8A* [2]. *SCN1A* and *SCN2A* are associated with a variety of epilepsy syndromes; most notably, Dravet syndrome is caused by an *SCN1A* mutation in over 80% of reported cases [2, 3]. Clinical features can help differentiate the genetic cause of seizures. For example, the mean seizure onset of 5 months old is similar for both Dravet syndrome and *SCN8A*-related encephalopathy, however, unlike Dravet syndrome the upper age limits in cases due *SCN8A* can be after one year [1, 4]. Epileptic spasms are seen with *SCN8A* mutations, but this is not a seizure-type associated with Dravet syndrome [1]. VGSC mutations result in clinical heterogeneity that is observed not only when comparing different mutations within the same gene, but even in different patients carrying the same mutation [1–4]. Detailed genotype-phenotype evaluations and *in vitro* functional studies can confirm pathogenicity and predict outcome or response to treatment. Clinical presentation and specific EEG features will likely be important for selecting patients for genetic testing.

Epileptic Encephalopathies are a heterogeneous group of severe epilepsy syndromes with onset in infancy and childhood associated with severe cognitive and behavioral disturbances. A genetic diagnosis for a patient with epileptic encephalopathy can aid with prognosis as well as treatment. For example, although seizures may be worsened by carbamazepine in Dravet syndrome, this anticonvulsant may be helpful in some patients with *SCN8A* [1, 4]. As the utilization of genetic testing in the diagnosis of epileptic encephalopathies increases, we can hope for improved outcomes through new therapeutic paradigms and personalized medicine.
